# Drug-Eluting Balloons Versus Conventional Balloon Angioplasty for Peripheral Pulmonary Artery Stenosis in Childhood

**DOI:** 10.1007/s00246-025-04047-5

**Published:** 2025-10-18

**Authors:** Timo J. Herling, Peter A. Zartner

**Affiliations:** Department of Pediatric Cardiology, German Pediatric Heart Centre, University Hospital of Bonn, Venusberg-Campus 1, Building 30, 53127 Bonn, Germany

**Keywords:** Pediatric cardiology, Drug-eluting balloons, Angioplasty, Peripheral pulmonary artery stenosis

## Abstract

The objective of this study was to evaluate the efficacy of drug-eluting balloons angioplasty (DEBA) compared with conventional balloon angioplasty (CBA) in children with peripheral pulmonary artery stenoses (PAS). PAS is a common complication in children with congenital heart defects (for example tetralogy of Fallot), postoperative patients, and certain syndromes (Alagille, Noonan, Williams–Beuren). A retrospective qualitative analysis was performed of the individual stenoses, which were treated by dilation via cardiac catheterization and controlled after several months. The study involved 103 stenoses from 2012 to 2022 whose diameters were measured at the initial and at follow-up catheterization. The intervention group (DEBA) was compared with the control group (CBA) in terms of lumen diameter change, hemodynamics, and infant development. During mean follow-up period of 10.2 ± 8.1 months, the diameter growth rate was significantly higher in patients treated with DEB (diameter growth/month: 0.151 ± 0.103 mm vs. 0.087 ± 0.032 mm, *p* =  < .001; fractional diameter growth/month: 4.1 ± 3% vs. 2.2 ± 1.2%, *p* =  < .001). The right ventricular load, which is represented by the ratio of systolic right ventricular pressure and systolic systemic arterial pressure, was significantly reduced in DEB-treated stenoses (RV-SA pressure ratio change: − 6.4 ± 12.4% vs. 4.3 ± 22.2%, *p* = .023). This study demonstrated that DEB for treatment of PAS was effective and resulted in a higher lumen increase of the affected vessel than the treatment with conventional balloons.

## Introduction

What is known:

Peripheral pulmonary artery stenosis in childhood is an obstructive disease that occurs in a heterogeneous morphologic spectrum. Clinical presentation can range from minor symptoms to signs of right ventricular decompensation.

Percutaneous transluminal angiography with balloon dilation is a proven therapy for lesions.

What the study adds:

Transluminal dilation using drug-eluting-balloons is superior to conventional form of angioplasty.

What the study could change:

The use of DEBs is more effective in the treatment of PAS than conventional therapy and can be used more frequently.

Peripheral pulmonary artery stenosis (PAS) is an obstructive lesion distal of the truncus pulmonalis. It can appear as an acquired disease or with several congenital heart defects such as the tetralogy of Fallot, pulmonary atresia or the dextro-transposition of the great arteries (d-TGA). In addition, it may occur in genetic syndromes such as Williams, Alagille, or Noonan [1]. Another possible cause is post-surgical stenosis. In this study, however, the children who had previously undergone surgery were excluded due to the difference in pathogenesis. Most of the patients are diagnosed in childhood with characterized symptoms as dyspnea, fatigue, tachycardia, and an abnormal perfusion. The peripheral pulmonary artery stenosis reduces cross sectional area, resulting in an elevated pressure load on the right ventricle. This can lead from right ventricular hypertension up to pulmonary heart disease with ventricular dysfunction [2]. There are several therapeutic options to increase the diameter of the involved vessel.

### Therapeutic Options

Percutaneous transluminal angioplasty (PTA) with low-pressure balloons in PAS was introduced in the 1980 s and became an accepted alternative to surgical therapy [3]. Subsequent development brought innovations such as high-pressure balloons, cutting balloons, stents, and DEBs. The simple angioplasty is still frequently used as first-stage therapy. The vessel diameter is increased by dilation, resulting in a decrease of pulmonary artery and right ventricular pressure [4]. Especially the low complication rate and the minimal risk in intervention is an advantage compared to the other options of treatment [5]. Stent placement is a commonly used option in vessels that are resistant to simple balloon angioplasty. Stents can be used in vessels that are large, more proximal or compressed by adjacent structures. Stents need to be redilated by the growth of the pulmonary vessels in early childhood [6, 7]. The general scheme for PAS treatment in our department is to start with balloon angioplasty in younger children and, as they grow, to apply stent placement [7].

### Main Problems

The reduced diameter of the vessels is usually of unknown pathogenesis. A possible cause is intimal proliferation. Insufficient growth of the lumen diameters and increased right ventricular load can be observed [8, 9].

### Drug-Eluting Balloons

In order to reduce complications such as intimal proliferation or idiopathic vascular stenosis, DEBs are increasingly used in many specialties [10, 11]. DEBs are balloons for angioplasty that are coated with an antiproliferative substance, most commonly paclitaxel. Paclitaxel is a lipophilic, antiproliferative cytostatic drug that leads to an irreversible arrest of cell division between meta- and anaphase. There are a few reasons why paclitaxel is a good candidate for local drug therapy. Paclitaxel is a very lipophilic drug, which allows it to be rapidly absorbed into cells. Moreover, due to its effect mechanism, it can achieve a long effect by a short application time [12, 13]. The coating technique of the balloons allows an immediate drug release during inflation [14]. There is only little research about systemic drug release with the use of DEBs. It turns out that DEBs releases drugs into the vessel wall as well as into the circulation during implantation. Studies showed that the serum level of paclitaxel over time was always significantly below the systemic effective level of 85 ng/mL. A case study reviewed serum levels following an application of 754-ug paclitaxel at dilation. The highest plasma level was reached after 4 h with 20.18 ng/mL, not even close to the systemic effective level. There were no adverse systemic events [15–17].

## Methods

### Patient Selection and Study Design

A total of 103 stenoses were evaluated in two separately blocks (CBA vs DEBA). The study is a conducted retrospective review of all dilated peripheral pulmonary artery stenoses in Bonn University Hospital between 2012 and 2022. The following inclusion criteria were set: The patient had at least two comparable catheter procedures, the first of which was the angioplasty intervention. The subsequent catheterization was rated as the first follow-up examination.

The study included all patients who had unilateral or bilateral PAS. Only patients were included if there is an intact ventricular septum and the stenotic vessel is a major vessel. Cardiac catheterization images from each patient were evaluated. PAS was diagnosed by invasive angiography. The decision to recanalize by balloon dilatation and to use CB or DEB was determined by the surgeon. DEBs were primarily used when the artery showed a tendency for restenosis, indicated by intimal proliferation. The hemodynamic, interventional, and clinical data were evaluated. Patients with an intervening corrective surgery (e.g., Fontan) or stent replacement were excluded from the study.

### Angiographic Analysis

The percutaneous transluminal angioplasty was performed with different CBs and DEBs (Table 1). Angiography was performed before and after all dilations and at follow-up catheterization. The identical projections and technical requirements were always used. The projections were calibrated for measurements. The vascular diameter was measured in the inner stenotic area. The Hyper.PACS system (Telemis) and the IntelliSpace Cardiovascular system (Philips) was applied for imaging and measurement of vascular diameters. For the evaluations and measurements, the individual angiographic recordings were blinded.

**Table 1:** Devices and specifics

	*n*	%	Diameter, mm	Length, mm
*CB*				
TYSHAK MINI® NuMED Inc., Hopkinton, NY	19	18.4	6.74 ± 1.73	20 ± 0
Powerflex® Cordis, Miami Lakes, FL	17	16.5	9.88 ± 1.65	21.18 ± 3.32
Armada™ Abbott, Lake County, IL	9	8.7	11.11 ± 1.05	20 ± 0
EMERGE™ Boston Scientific, Marlborough, MA	4	3.9	5.25 ± 0.5	17.5 ± 2.89
Total	49	47.6	8.51 ± 2.51	20.2 ± 2.27
*DEB*				
ELUTAX-SV Aachen Resonance, Aachen, GER	32	31.1	7.84 ± 1.85	23.13 ± 7.38
Advance-PTX® Cook Medical LLC, Bloomington, IN	14	13.6	7.29 ± 0.83	20 ± 0
IN.PACT™ Medtronic, Minneapolis, MN	6	5.8	5.83 ± 1.17	33.33 ± 10.33
Ranger™ Boston Scientific, Marlborough, MA	2	1.9	6.50 ± 2.12	30 ± 0
Total	54	52.4	7.43 ± 1.68	23.7 ± 7.6
All	103	100		

### Follow-Up and Definition

Patients with PAS receive routine follow-up. Depending on clinical symptoms and examination results, cardiac catheterization was performed. The follow-up angiography was conducted using identical projections and analyses. Angiographic lumen growth (difference between the pre-procedural and follow-up lumen diameter) was the primary endpoint of this study. Due to the different follow-up intervals, the calculation was made for the comparison in absolute lumen growth rate (mm/month) and fractional lumen growth rate (%/month).

### Statistical Analysis

Analysis of the data was performed according to intention-to-treat. Data were tabulated and summarized as mean ± SD. The *p* values were adjusted according to Wilcoxon rank-sum test of combining independent tests and the *t* test for independent tests. *P* value of < 0.05 was considered significant.

Calculation of the pressure values were based on following measurements at initial and follow-up catheterization: systolic right ventricular pressure (RV1 & RV2), systemic arterial pressure (SA1 & SA2), and RV-SA pressure ratio (RV/SA). The ratio changes were analyzed using a *t* test for independent groups to evaluate the differences between lesions treated with DEB and CB. The effect size was calculated as Cohen’s d. To assess the equality of variances in lumen diameter findings the Levene’s test was used (Table 2). Balloon-to-stenosis ratio was calculated as the maximum balloon diameter (mm) divided by the stenosis diameter (mm) for each treated lesion. Group comparisons of the balloon-to-stenosis ratio were performed with an the *t* test for independent tests because the variable was normally distributed.

**Table 2:** Lumen diameter findings Interval to follow-up >  = 1 month

	Conventional balloon (*n* = 44)	Drug-eluting balloon (*n* = 52)	p Value
Interval to follow-up (month)	12.0 ± 7.8	10.0 ± 8.1	.919^a^
Diameter pre 1 st intervention (mm)	4.60 ± 1.74	3.97 ± 1.23	.018^a^
Balloon diameter to stenosis ratio	2.05 ± 0.73	1.97 ± 0.51	.503^b^
Fractional diameter growth during 1 st dilation (%)	24.16 ± 7.14	24.47 ± 9.95	.858^b^
Diameter at follow-up (mm)	5.60 ± 1.70	5.04 ± 1.53	.612^a^
Absolute lumen growth (mm)	1.00 ± 0.67	1.06 ± 0.57	.273^a^
Fractional lumen growth (%)	28 ± 27	28 ± 15	.002^a^

Values are mean ± SD. The *p* values were adjusted to the Levene’s test^a^ to assess the equality of variances and the *t* test^b^ of combining independent tests

Luminal diameter changes until the follow-up catheterization were based on following values: minimal diameter at initial angiography (diameter1), minimal diameter at follow-up-angiography (diameter2), absolute lumen growth (diameter2-diameter1), fractional lumen growth ([diameter2-diameter1]/diameter1), absolute lumen growth rate in mm/month ([diameter2-diameter1]/time) and as fractional lumen growth rate in percentage/month (fractional lumen growth/time). A Wilcoxon rank-sum test of combining independent tests was used to compare lumen changes in each balloon type (CB vs. DEB), with *p* value of < 0.05 considered statistically significant. To avoid measurement errors in the case of minor differences in diameter, follow-up periods < 1 month were excluded from analysis. In order to estimate the effect size, the Wilcoxon signed-rank test (WSR) with the Pearson correlation coefficient (r) was used.

To examine whether the dilation immediately after the balloon angioplasty differs between the comparison groups, the diameter before and after dilation were measured. The increase in diameter was calculated as fractional growth and subsequently analyzed for statistical significance using the *t* test for independent tests, provided the results followed a normal distribution.

In addition, an analysis of the weight development of newborns, infants, and toddlers (age 0–36 months) was also carried out. Patients with certain genetic syndromes (Alagille, Noonan, Williams–Beuren) were excluded from this analysis. For this evaluation, the change in weight between the examinations was calculated as the monthly weight gain rate ([weight2-weight1]/interval). The *t* test for independent tests was used to examine the differences between the intervention groups in terms of weight gain rate difference.

## Results

A total of 103 lesions were treated by balloon angioplasty during the study, consisting of 34 patients who were treated by 49 CBs and 29 patients who received 54 DEBs. There were 13 patients who had bilateral stenoses and 25 patients who had repetitive angioplasty. Table 3 shows the baseline clinical data at first intervention.

**Table 3:** Baseline clinical data and procedural data

	Conventional balloon (*n* = 49)	Drug-eluting balloon (*n* = 54)
Gender (F:M)	19 (39): 30 (61)	30 (56): 24 (44)
Age at 1 st cath (months)	42.0 ± 42.8	44.4 ± 36.1
Weight at 1 st cath (kg)	14.0 ± 9.1	14.4 ± 8.2
Height at 1 st cath (cm)	91.2 ± 26.3	94.2 ± 22.5
RV pressure (mmHg)	67 ± 23 (*n* = 35)	83 ± 22 (*n* = 38)
SA pressure (mmHg)	87 ± 17 (*n* = 34)	91 ± 16 (*n* = 32)
Treated vessel		
LPA	25 (51)	33 (56)
RPA	24 (49)	24 (44)
Balloon size		
Diameter (mm)	8.5 ± 2.5	7.4 ± 1.7
Length (mm)	20.2 ± 2.3	23.7 ± 7.6

Values are mean ± SD or n (%)

*RV* right ventricular, *SA* systemic arterial

### First Angioplasty

The decision to catheterize was made based on clinical examinations and medical imaging. Whether an angioplasty is necessary was decided during the intervention. The goal of treatment is to eliminate the stenosis, to enable the side-by-side equal lung blood circulation and to reduce the right ventricular pressure [18–20]. Table 1 shows the balloons that were used in our review. To compare whether the use of different balloons varied over the study time, the median test for independent tests was applied. The median showed no statistically significant difference between the study groups (*p* = 0.143). Balloon-to-stenosis ratios were normally distributed. The mean balloon-to-stenosis ratio was 1.97 ± 0.51 in the DEB group and 2.05 ± 0.73 in the CB group; the difference was not significant (*t* test, *p* = 0.503).

#### Conventional Balloons

**Fig. 1:**
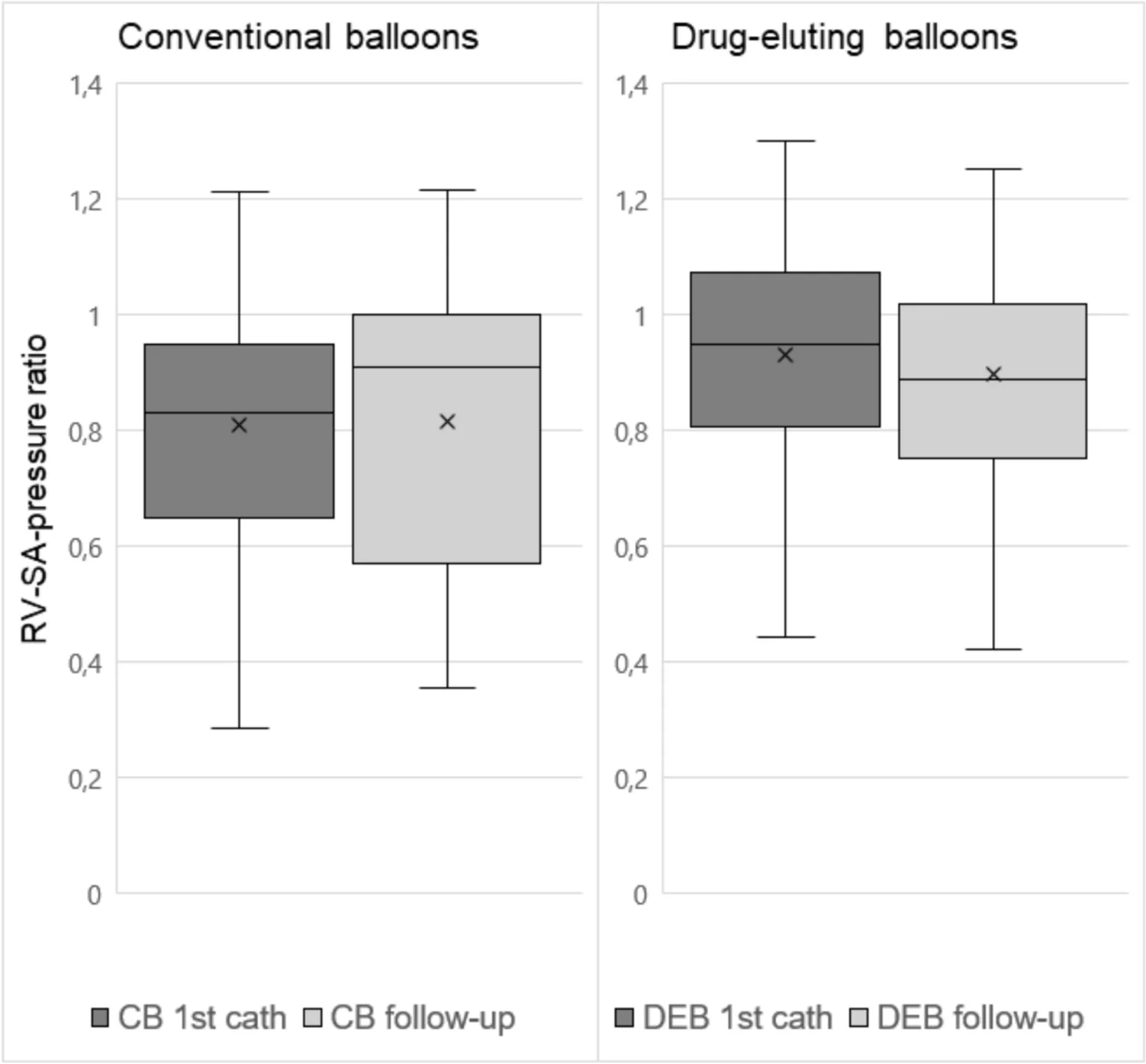
Hemodynamic findings

A total of 49 lesions were dilated with CB (balloon diameter 8.51 ± 2.51 mm). The average systolic right ventricular pressure was 67 ± 23 mmHg, and the systemic arterial pressure was 87 ± 17 mmHg before the first intervention. The average pressure ratio (RV/SA) was 81 ± 30% (Fig. 1). Minimum lesion diameter was 4.60 ± 1.74 mm at first catheterization (Table 2). Average weight at first intervention was 8.7 ± 5.9 kg. The group includes 7 patients who had bilateral stenoses.

#### Drug-Eluting Balloons

There were 54 lesions treated with DEB (balloon diameter 7.43 ± 1.68 mm). DEBs procedures were performed with a mean inflation time of 37 ± 8 s (*n* = 41). The average systolic right ventricular pressure was 83 ± 22 mmHg, and the systemic arterial pressure was 91 ± 16 mmHg before the first intervention. The average pressure ratio (RV/SA) was 92 ± 16% (Fig. 1). Minimum lesion diameter was 3.97 ± 1.23 mm at first catheterization (Table 2). Average weight was 8.5 ± 2.5 kg. The group includes 8 patients who had bilateral stenoses.

### Follow-Up Catheterization

The first catheterization after the initial one with visualization of the pulmonary arteries was considered as follow-up. A left heart examination was not necessarily performed, which made it impossible to invasively measure systolic arterial pressures in all of them. Whether interventions were performed during the follow-up examination was not considered in this study.

#### Conventional Balloons

Follow-up duration of the 44 CBA-treated lesions was an average of 12.0 ± 7.8 months. Mean RV pressure was 68 ± 23 mmHg, SA pressure was 83 ± 13 mmHg, and RV/SA ratio was 82 ± 25% (Fig. 1). Minimum lesion diameter was 5.60 ± 1.70 mm at follow-up. Absolute lumen growth was 1.00 ± 0.67 mm, fractional lumen growth was 28 ± 27% (Table 2). Average weight at follow-up was 10.2 ± 6.1 kg. To investigate whether the use of high-pressure balloons (i.e., Powerflex) versus low-pressure balloons (i.e., Tyshak mini) affects effectiveness, no significant difference is observed in normally distributes results (diameter change per month in low-pressure balloons = 0.79 ± 0.03 mm/month vs diameter change per month in high-pressure balloons = 0.92 ± 0.03 mm/month). This was analyzed using a *t* test for independent test (*p* = 0.184).

#### Drug-Eluting Balloons

The average follow-up duration for the 52 DEB-treated lesions was 10.0 ± 8.1 months. Mean RV pressure was 81 ± 20 mmHg, SA pressure was 92 ± 16 mmHg, and RV/SA ratio was 90 ± 27% (Fig. 1). Minimum lesion diameter was 5.04 ± 1.53 mm at follow-up. Absolute lumen growth was 1.06 ± 0.57 mm, fractional lumen growth was 28 ± 15% (Table 2), and average weight was 9.7 ± 2.5 kg.

### Comparison of CBA Versus DEBA Lumens at Follow-Up Catheterization

Table 4 shows the most statistically relevant data for comparing the two interventions groups.

**Table 4:** Comparison of DEB versus CB in diameter, hemodynamics, and weight gain

	Conventional balloon	Drug-eluting balloon	*p* value	Effect size
RV-SA pressure ratio change (%)	4.3 ± 22.2 (*n* = 23)	− 6.4 ± 12.4 (*n* = 24)	.023^b^	d:.598
Absolute lumen growth rate (mm/month)	0.087 ± 0.032 (*n* = 44)	0.151 ± 0.103 (*n* = 52)	<.001^a^	r: 0.34
Fractional lumen growth rate (%/month)	2.2 ± 1.2 (*n* = 44)	4.1 ± 3 (*n* = 52)	<.001^a^	r: 0.39
Weight gain rate (kg/month) Age < 3 years	0.14 ± 0.13 (*n* = 17)	0.27 ± 0.25 (*n* = 17)	.032^b^	d:.661

Values are mean ± SD. The *p* values were adjusted to the Wilcoxon rank-sum test^a^ and the *t* test^b^ of combining independent tests

The RV-SA pressure ratio changes of the two study groups without a septal defect were statistically normally distributed. The *t* test for independent tests was used to evaluate differences in RV-SA pressure ratio changes (Fig. 1). In this analysis, only patients without septum defect were included (CB *n* = 23; DEB *n* = 24). Ratio change is significantly different when using DEBs (M =  − 6.4; SD = 12.4) in comparison to the use of CBs (M = 4.3; SD = 22.2), t(45) = 2.048, *p* < 0.023, d = 0.598.

When examining fractional diameter growth immediately after the intervention, no significant difference was found between CBs and DEBs using the *t* test for independent tests (M = 24.16; SD = 7.14) vs. (M = 24.47; SD = 9.95), t(96) = −0.179, *p* = 0.858 (Table 2).

**Fig. 2:**
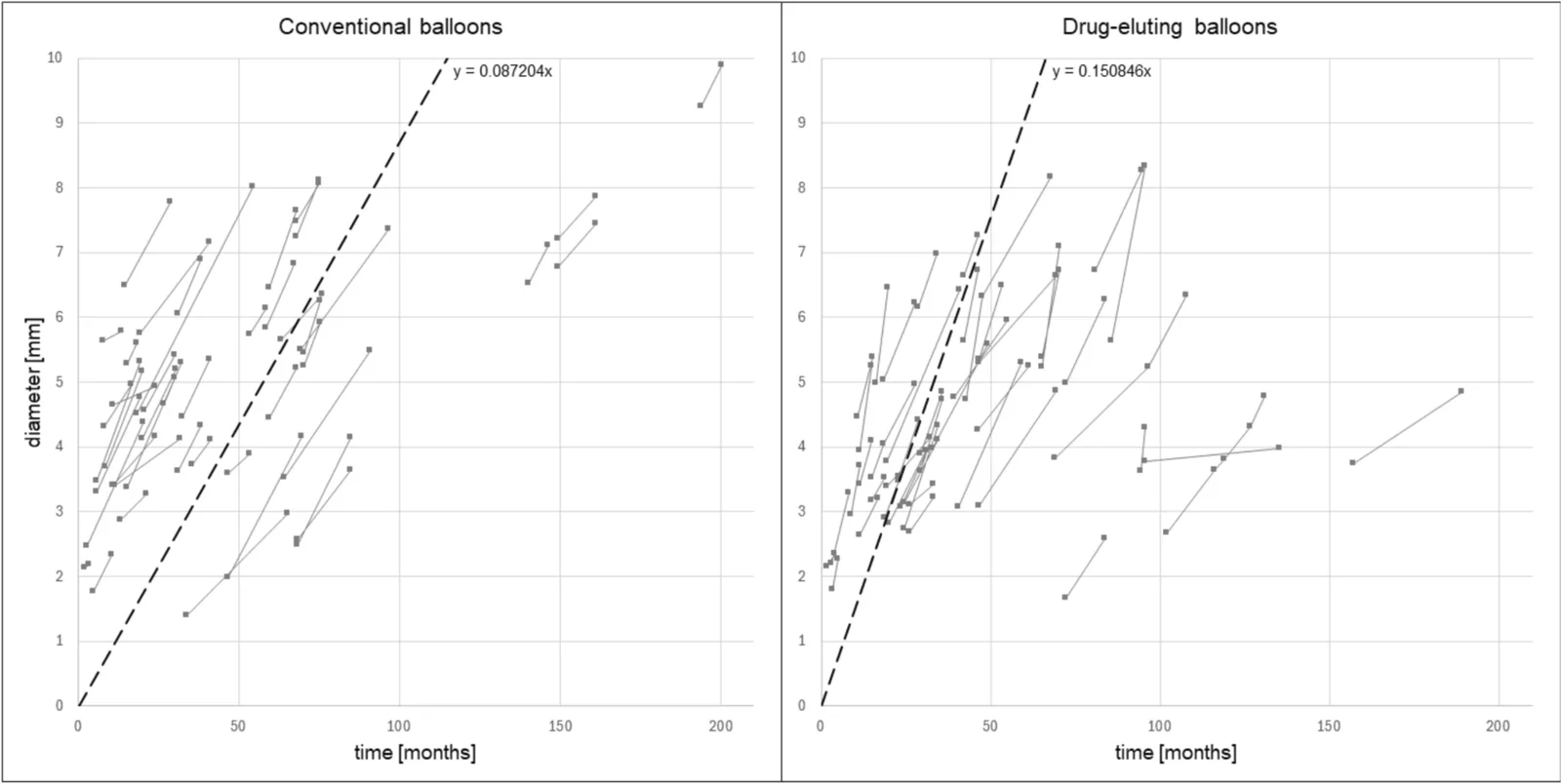
Diameter change

To consider the different follow-up periods, lumen diameter changes were converted to diameter changes per month. Absolute lumen growth per month was 0.087 ± 0.032 mm and fractional lumen growth per month was 2.2 ± 1.2% in the CB group. In the DEB group absolute lumen growth per month was 0.151 ± 0.103 mm and fractional lumen growth per month was 4.1 ± 3%. The distributions differed between both groups, Kolmogorov–Smirnov *p* < 0.05. Using Wilcoxon rank-sum test of combining independent tests, lesions in which DEB (M = 0.151; SD = 0.103) were applied had a significantly higher absolute lumen growth rate compared to when CB (M = 0.087; SD = 0.032) were used, U = 691, Z =  − 3.331, *p* < 0.001, r = 0.34. DEB-treated (M = 4.1; SD = 3) lesions had a significantly higher fractional lumen growth rate than CB-treated (M = 2.2; SD = 1.2) lesions, U = 626, Z =  − 3.809, *p* < 0.001, r = 0.39. Figure 2 shows individual diameter changes and the resulting trend line.

The values of the weight gain per month were normally distributed. Therefore, the *t* test for independent tests was applied. Patients treated with DEB (M = 0.27; SD = 0.25) had a significantly higher weight gain rate than those treated with CB (M = 0.14; SD = 0.13), t(32) = 1.926, *p* < 0.032, d = 0.661.

## Discussion

PAS of heterogeneous pathogenesis usually require intervention. Further reinterventions often become necessary during development. The first and safest therapy is often angioplasty, in which the stenosis can be effectively removed, and no foreign body remains. Another possible effective and permanent option is the implantation of stents. Particularly in young children, it is often not possible to implant a stent due to the anatomic conditions and changes. Due to the growth of the pulmonary vessels, redilation or surgical removal of the stent becomes necessary [6, 8, 19–22]. This shows the importance of balloon angioplasty to treat the stenosis itself and, if necessary, to bridge the time interval until stent implantation [19, 21–23]. In order to make balloon dilation as effective as possible, we are increasingly using DEBs and analyzed the effectiveness of DEB versus CB in this study. Never before has such a large number of PAS and its treatment using angioplasty been analyzed.

Our study demonstrated that DEB for treatment of PAS was effective and resulted in a higher lumen increase of the affected vessel than the treatment with conventional balloons. Previous studies have shown the significant effect of DEB in various vascular diseases, such as coronary artery disease, atretic pulmonary veins, and femoropopliteal artery disease [10, 11, 24, 25]. Conventional balloon angioplasty as treatment for vascular stenoses is limited by insufficient growth and restenosis. The complication of PAS appears to be related to myofibroblast proliferation [3, 4, 8]. Therapy targeting myofibroblast proliferation in the form of Paclitaxel was used. This has the advantage of achieving a precise biochemical and local effect to prevent neointimal proliferation in the stenotic area. In our study, we did not measure serum levels of the cytostatic drug paclitaxel. Müller et al. [15] measured low concentrations of paclitaxel after balloon dilation with DEB. It can therefore be assumed that there are no side effects of a systemic therapy because of the sub-therapeutic serum level.

Compared with CBA, DEBA exhibited a significant higher lumen growth rate by the time to follow-up. In addition, DEB was associated with a significant reduction of the right ventricular load. We found that the use of DEB was associated with increased weight gain at age between birth and 3 years. There is very little data concerning the effects of DEB on PAS in childhood. In this study, we found an almost 60% higher average growth rate of the lumen. Compared to the pressure values before the first intervention, the right ventricular load was considerably lower with the use of DEB. Our study suggests that DEB could be the more effective choice for the therapeutic option of angioplasty. The concept of the DEB avoids or delays the implantation of a stent, which would be an alternative. It is also possible to reduce the dual antiplatelet therapy, in contrast to the implantation of stents [26].

### Study Limitations

Limitations of this study are the limited number of stenoses and the fact that it is retrospective. The study is also a single-center trial which selects conventional balloon angioplasty as comparison. Peripheral pulmonary artery stenosis shows wide morphological variability across patients, and this heterogeneity may limit the generalizability of our findings. Pre- and post-stenosis vessel diameters were not systematically recorded during the study period. To mitigate these issues, we report balloon-to-stenosis ratios and immediate percent lumen increase (which were comparable between groups), and we advise that future prospective studies standardize diameter measurements and allocation protocols. Furthermore, only individual stenoses and their progression to the follow-up can be compared. Due to the fact that our patients often undergo multiple dilations with different balloons throughout their development, it was not possible to compare the study groups in long-term follow-up. The differences in actual measurements are small over the short follow-up period. Further study is needed to determine whether these will be significantly significant. We focused especially on lumen growth between initial and follow-up catheterization, another study should examine the long-term effects and patient survival. In this retrospective study, we analyzed only CBs and DEBs. Further studies needed to compare these findings with the use of cutting balloons.

### Conclusion

This single-center retrospective study suggests that DEB contribute significantly improved angiographic and hemodynamic outcomes than CB in children with PAS. Balloon angioplasty is indispensable in the treatment of PAS. DEBs are used to make the angioplasty as effective and sustainable as possible. This study shows that the established procedure of angioplasty with DEBs in other medical specialties is also a suitable candidate for the treatment of PAS.

## Data Availability

No datasets were generated or analysed during the current study.

## Abbreviations

CB: Conventional balloon
CBA: Conventional balloon angioplasty
DEB: Drug-eluting balloon
DEBA: Drug-eluting balloon angioplasty
PAS: Peripheral pulmonary artery stenosis
